# Paradox Elimination in Dempster–Shafer Combination Rule with Novel Entropy Function: Application in Decision-Level Multi-Sensor Fusion

**DOI:** 10.3390/s19214810

**Published:** 2019-11-05

**Authors:** Md Nazmuzzaman Khan, Sohel Anwar

**Affiliations:** Department of Mechanical and Energy Engineering, IUPUI, Indianapolis, IN 46224, USA; soanwar@iupui.edu

**Keywords:** Dempster–Shafer evidence theory (DST), uncertainty measure, novel belief entropy, multi-sensor data fusion, decision-level sensor fusion

## Abstract

Multi-sensor data fusion technology in an important tool in building decision-making applications. Modified Dempster–Shafer (DS) evidence theory can handle conflicting sensor inputs and can be applied without any prior information. As a result, DS-based information fusion is very popular in decision-making applications, but original DS theory produces counterintuitive results when combining highly conflicting evidences from multiple sensors. An effective algorithm offering fusion of highly conflicting information in spatial domain is not widely reported in the literature. In this paper, a successful fusion algorithm is proposed which addresses these limitations of the original Dempster–Shafer (DS) framework. A novel entropy function is proposed based on Shannon entropy, which is better at capturing uncertainties compared to Shannon and Deng entropy. An 8-step algorithm has been developed which can eliminate the inherent paradoxes of classical DS theory. Multiple examples are presented to show that the proposed method is effective in handling conflicting information in spatial domain. Simulation results showed that the proposed algorithm has competitive convergence rate and accuracy compared to other methods presented in the literature.

## 1. Introduction

Multi-sensor fusion means the combination of information from multiple sensors (homogeneous or heterogeneous) in a meaningful way so that we can overcome any limitations inherent to a single sensor or information source. Based on the identified strengths and weaknesses of previous work, a principled definition of information fusion is proposed in Reference [1]: “Information fusion is the study of efficient methods for automatically or semi-automatically transforming information from different sources and different points in time into a representation that provides effective support for human or automated decision making.” A multi-sensor system has two distinct advantages over a single sensor system when used with a proper fusion algorithm:

- A single sensor may provide faulty, erroneous results, and there is no way to modify that other than by changing the sensor. A multi-sensor system provides results with diverse accuracy. With the help of a proper fusion algorithm, faulty sensors can be easily detected.
- A multi-sensor system receives information with wide variety and characteristics. Thus, it helps to create a more robust system with less interference.

**Figure 1:**
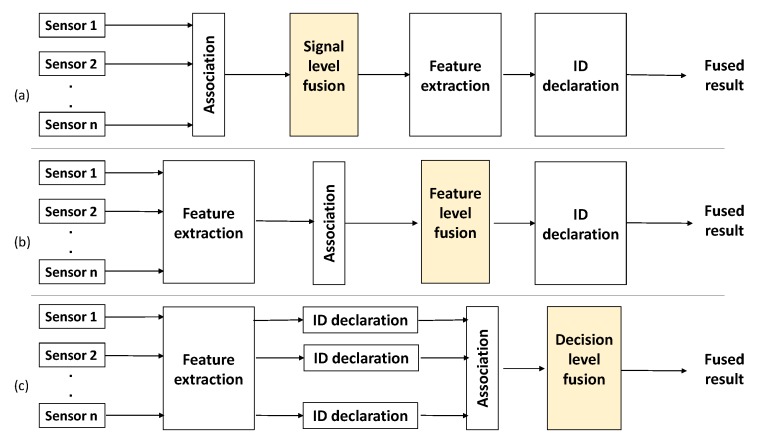
Data fusion at three different levels: (**a**) Signal-level fusion, (**b**) feature-level fusion, and (**c**) decision-level fusion.

Now, to combine inputs from different sensors at the decision level to achieve correct object classification, we need a robust decision-level sensor-fusion algorithm. As shown in Figure 1 [2], sensor fusion can be represented at three different levels. The signal level can be explained if raw pixels from multiple cameras are combined. Feature-level sensor fusion can be explained by the following. Hand-coded features (area or moment of certain object) are extracted from images. Then, features are fused using some clustering algorithm. The final decision is made using the output of the clustering algorithm. At the decision level, a decision is already made by using a supervised/unsupervised algorithm. Then, decisions from multiple sensors are fused using Bayes or another information fusion algorithm. At the decision level, a crucial issue in multi-source information fusion is how to represent and determine the imprecise, fuzzy, ambiguous, inconsistent, and even incomplete information [3]. As a tool to manipulate an uncertain environment, Dempster–Shafer (DS) evidence theory is an established system for uncertainty management [4,5]. The limitations of the original DS combination rule and works to eliminate them are discussed in Section 4.

## 2. Dempster–Shafer Evidence-Based Combination Rule

### 2.1. Frame of Discernment (FOD)

The frame of discernment contains M mutually exclusive and exhaustive events (also represented by X in this research).

(1) $$X\;=\;\Theta \;=\;\{\theta_{1},\theta_{2},\ldots .,\theta_{M}\}$$

The representation of uncertainties in the DS theory is similar to that in conventional probability theory and involves assigning probabilities to the space Θ. However, the DS theory has one significant new feature: it allows the probability to be assigned to subsets of Θ as well as the individual element $\theta_{i}$. Accordingly, we can derive the power set $2^{\Theta }$ of DS theory:

(2) $$2^{\Theta }\;=\;\left\{\phi ,\left\{\theta_{1}\right\},\left\{\theta_{2}\right\},\ldots .,\left\{\theta_{1},\theta_{M}\right\},\ldots .,\Theta \right\}$$

where ϕ is an empty set. It is clearly seen in Equation (2) that the power set $2^{\Theta }$ has $2^{M}$ propositions. Any subset except singleton of possible values means their union. For example, $\left\{\theta_{1},\theta_{2},\theta_{3}\right\}\equiv \left\{\theta_{1}\cup \theta_{2}\cup \theta_{3}\right\}$. Complete probability assignment to a power set is called basic probability assignment (BPA).

### 2.2. Basic Probability Assignment (BPA)/Mass Function

Evidences in DS theory are acquired by multi-sensor information. Mass function (mass) is a function, $m:2^{\Theta }\to \left[0,1\right]$ that satisfies Equations (3) and (4):

(3) m(ϕ)=0

(4) $$\sum \{m\left(\theta \right)\forall \theta \in 2^{\Theta }\}$$

*m* is called basic probability assignment. Elements of power set having m(θ)>0 are called focal elements. This can be explained with the help of a simple example. Let the three objects to be detected be, Θ={a,b,c}. Powerset $2^{\Theta }\;=\;2^{3}\;=\;\left\{\phi ,a,b,c,\left\{a,b\right\},\left\{a,c\right\},\left\{b,c\right\},\Theta \right\}$. From a sensor or by an expert, the following mass values are assigned, m(a)=0.2,m(b)=0.3,m(a,b)=0.4,m(a,b,c)=0.1. The four subsets are called focal elements.

### 2.3. Dempster–Shafer Rule of Combination

The purpose of data fusion is to summarize and simplify information rationally, obtained from independent and multiple sources. DS combination rule emphasizes the agreement between multiple sources and ignores all the conflicting evidences through normalization. Any two mass functions B and C over the same FOD with at least one focal element in common can be combined into a new mass function using DS combination rule. The combination of two mass functions can also be said to take the orthogonal sum ⊕. The combination of two belief functions, the DS combination rule for combining two evidences $m_{1}$ and $m_{2}$, is defined as follows:

(5) $$m_{12}\left(A\right)\;=\;\frac{\sum_{B\cap C}\left\{m_{1}\left(B\right).m_{2}\left(C\right)\right\}}{1-K}$$

when A≠ϕ and m(ϕ)=0.

(6) $$K\;=\;\sum_{B\cap C\;=\;\phi }\left\{m_{1}\left(B\right).m_{2}\left(C\right)\right\}$$

where *K* is the degree of conflict in two sources of evidences. The denominator (1−K) is a normalization factor, which helps aggregation by completely ignoring the conflicting evidence and is calculated by adding up the products of BPAs of all sets where intersection is null. DS combination rule in Equation (5) conforms to both commutative law and associate law.

$$m_{1}⊕m_{2}\;=\;m_{2}⊕m_{1}$$

$$\left(m_{1}⊕m_{2}\right)⊕m_{3}\;=\;m_{1}⊕\left(m_{2}⊕m_{3}\right)$$

### 2.4. Belief and Plausibility Function

Given a basic assignment m, we can define a belief function Bel: $m:2^{\Theta }\to \left[0,1\right]$, such that for any A⊂Θ:

(7) $$Bel\left(A\right)\;=\;\sum_{B\subseteq A}\left\{m\left(B\right)\right\}$$

*Bel (A)* measures the belief that the element is a member of *A*. *m(A)* measures the amount of belief that one commits exactly to *A* alone; *Bel (A)* measures the total belief that the special element is in *A*. Based on the same premise, we have the following:

(8) $$Pl\left(A\right)\;=\;1-Bel\left(\bar{A}\right)$$

Pl(A) measures the degree to which one fails to doubt *A*. Pl(A) measures the total belief mass that can move into *A*, whereas Bel(A) measures the total belief mass that is constrained to *A*.

**Example** **1.** *Given, m(A) = 0.48, m(B) = 0.24, m(C) = 0.08, and m(Θ)=0.2,*

*Bel (A,B) = m(A) + m(B) + m(A,B) = 0.48 + 0.24 = 0.72*

*Pl (A,B) = m(A) + m(B) + m(A,B) + m(A,C) + m(B,C) + m(A,B,C) = 0.48 + 0.24 +0.2 = 0.92.*

Similarly, *Bel* and *Pl* values can be calculated for all the BPA, which is shown in Table 1.

## 3. Paradoxes (Source of Conflicts) in DS Combination Rule

Dempster–Shafer theory, introduced and developed by Dempster and Shafer [6,7,8], has many merits by contrast to Bayesian probability theory [9]. However, to use DS sensor fusion algorithm for robust application, we have to overcome the fusion paradoxes. Based on the application in a multi-sensor system, this theory also has its shortcomings [10]. The different levels of performance of sensors, cluster, and interference of a complex environment may lead to conflicts among evidences. When evidences are highly conflicting, the fusing results obtained by the DS combination method are normally contrary to common sense. When the conflicting factor *K* is close to 1, this rule cannot obtain reasonable fusing results as the denominator is approximately 0. These counterintuitive phenomena of the DS theory are called paradoxes. According to Reference [11], there are mainly three types of paradoxes.

### 3.1. Completely Conflicting Paradox:

In this situation, there are two sensors and one sensor output completely contradicts the other sensor output. The following example depicts the situation:

**Example** **2.** *In the multi-sensor system, assume that there are four evidences in the frame, that Θ={A,B,C}, and that proposition A is true.*

*Sensor 1: $m_{1}$(A) = 1, $m_{1}$(B) = 0, $m_{1}$(C) = 0,*

*Sensor 2: $m_{2}$(A) = 0, $m_{2}$(B) = 1, $m_{2}$(C) = 0,*

Here, the two sensors are completely conflicting each other. The conflicting factor in Equation (6) is *K* = 1, which reports that evidences from sensor 1 and sensor 2 are completely conflicting. Under such circumstances, the DS combination rule cannot be applied.

### 3.2. “One Ballot Veto” Paradox

For a multi-sensor system (more than two sensors), one sensor completely contradicts all other sensor outputs. The following example depicts the situation:

**Example** **3.** *In the multi-sensor system, assume that there are four evidences in the frame, that Θ={A,B,C}, and that proposition A is true.*

*Sensor 1: $m_{1}$(A) = 0.7, $m_{1}$(B) = 0.2, $m_{1}$(C) = 0.1,*

*Sensor 2: $m_{2}$(A) = 0, $m_{2}$(B) = 0.9, $m_{2}$(C) = 0.1,*

*Sensor 3: $m_{3}$(A) = 0.75, $m_{3}$(B) = 0.15, $m_{3}$(C) = 0.1,*

*Sensor 4: $m_{4}$(A) = 0.8, $m_{4}$(B) = 0.1, $m_{4}$(C) = 0.1,*

Clearly, sensor 2 is faulty and contradicts the results of the other 3 sensors. Applying DS combination rule, we get *K* = 0.9, $m_{1234}\left(A\right)$ = 0/0.1 = 0, $m_{1234}\left(B\right)$ = 0.097/.1 = 0.97, and $m_{1234}\left(C\right)$ = 0.003/0.1 = 0.03. The fusing results are contrary to the assumed proposition that *A* is true. A high value of *K* proposes high contradiction among sensors. This counterintuitive result is caused by the erroneous sensor 2 values. Interestingly, DS combination rule completely omits a proposition even if a single sensor outputs zero evidence.

### 3.3. “Total Trust” Paradox

Here, one sensor highly contradicts the other sensor but both of them have a common focal element with low evidence. The following example depicts the situation:

**Example** **4.** *In the multi-sensor system, assume that there are two evidences in the frame and that Θ={A,B,C}.*

*Sensor 1: $m_{1}$(A) = 0.95, $m_{1}$(B) = 0.05, $m_{1}$(C) = 0,*

*Sensor 2: $m_{2}$(A) = 0.0, $m_{2}$(B) = 0.1, $m_{2}$(C) = 0.9,*

Applying DS combination rule, we get $m_{12}\left(A\right)$ = 0, $m_{12}\left(B\right)$ = 1, and $m_{12}\left(C\right)$ = 0, *K* = 0.99. Here, common sense suggests that either m(A) or m(C) is correct, but the wrong proposition *B* is identified to be true with total confidence even though senor 1 and 2 nearly negates this idea.

## 4. Eliminating the Paradoxes of DS Combination Rule

Existing modified methods are divided mainly into three categories:

### 4.1. Modification of DS Combination Rule

Smet’s rule [12] is essentially the Dempster rule applied in Smet’s Transferable Belief Model. Smet believed that conflict is caused by incompleteness of frame of discernment Θ and moved mass of conflict directly to ϕ as an unknown proposition. This model is a slightly different formulation of DS theory, but the ideas are essentially the same. In Yager’s rule [13], the mass associated with conflict is directly given to universal set Θ. Yager’s rule provides the same results when conflict is zero. Although these two methods solve the conflict situation theoretically, the uncertainty of the system still exists. Bicheng et al. [14] modified Yager’s rule and conflicting probability of the evidences are distributed to every proposition based on average support. Inagaki [15] defined a continuous parameter class of combination operations, which subsumes both DS and Yager’s rule. Depending on conflict of information, his combination rule changed between DS and Yager combination rule. However, based on experience, if an engineer applied a weighting factor to one of the sensors credibility, this rule cannot be applied. Zhang [16] pointed out that DS rule fails to take into account the focal element intersection. He presented the “two frame” representation of DS theory, where he measures focal element intersections based on cardinality. Li [17] used the interaction between focal elements and proposed two weighted redistribution methods, which consider the associative relationship among the evidences collected from multi-sources. His argument was that, if a body of evidence is greatly supported by others, this piece of evidence should be more important and has great effect on the final combination results. On the contrary, if a body of evidence is highly conflicting with others, this piece of evidence should be less important and has little effect on the final combination results. However, all these methods sometimes violate the theoretical properties of DS combination rule like commutativity and associativity.

### 4.2. Revision of Original Evidence before Combination

Commutative and associative properties of DS rule are important for multi-sensor information fusion, which may get lost when the original rule is tampered with. As a result, the propositions are modified so that conflict among the evidences are resolved before applying them in DS combination rule. Chen et al. [18] used triangular functions to set a fuzzy model for each sensor. Assuming each sensor output is gaussian, BPA was determined from the sensor outputs using the fuzzy model. Then, the raw BPA was weighted using the credibility of each BPA before fusing. Sun [19] also used fuzzy membership function to convert sensor values to fuzzy values. Support degree was calculated using an error distance function. If sensor output is not gaussian, then fuzzy set methods cannot be applied. Instead of distance function, an entropy function (Deng entropy [9]) was used to calculate the credibility of evidence in Reference [20]. This was inspired by Murphy’s method [21], which used an average of BPAs. Murphy’s method had a fast convergence rate but failed to consider the relation between focal elements. Jiang [22] used an entropy function to measure the weight of the evidence to modify them before applying to DS rule. Xiao [23] used almost the same procedure as Jiang but with a different distance function to measure the credibility. Murphy’s method is the simplest to implement, and most of the methods within this type are inspired by his method.

### 4.3. Hybrid Technique Combining Both Modification of DS Rule and Original Evidence

Through the comparison between two kinds of conflict resolutions, it is easy to see the underlying logic of two methods. Method 1 cancels the normalization step in DS theory and redistributes the conflict with different measure. Method 2 considers the essential differences between propositions of each sensor in multi-sensor systems and solves the conflict by modifying the original evidence. If methods 1 and 2 are combined, then the inherent paradoxes of DS rule are solved. Building on this idea, Lin et al. [24] and Ye Fang et al. [11] published several new improvements of original DS combination rule. They improved the fusion results, but the results were often too complicated and overengineered to apply for real-time use. These methods also lose commutative and associative properties of DS rule.

How to accurately measure the conflicting evidences under DS framework is still an open issue. Keeping the commutative and associative properties of the original DS combination rule and eliminating the paradoxes are critical for multi-sensor fusion. There is still room for improvement to properly measure the conflicts between evidences and to obtain appropriate weights for each evidence. Based on this, an improved combination method is proposed which follows “revision of original evidence before combination” method. A novel entropy function is proposed which can better capture the conflicts between evidences. Reward and penalty are imposed on evidences based on how they agree or disagree with each other. The amount of reward or penalty is determined by the entropy function. Then, the modified weight value (reward or penalty) is applied in adjusting the body of the evidences before using the Dempster’s combination rule (n−1) times, when there are *n* number of evidences (sensors). The simulation experiments illustrate that the proposed method is reasonable and efficient in coping with the conflicting evidences.

## 5. Entropy in Information Theory under DS Framework

Information is a measure of the compactness of a distribution; logically, if a probability distribution is spread evenly across many states, then its information content is low, and conversely, if a probability distribution is highly peaked on a few states, then its information content is high [25]. Information is a function of distribution. Entropy measures the compactness of a distribution of information. Entropy is zero when BPA is assigned to a single element, thus creating the most informative distribution. When BPA is uniformly distributed, entropy is at maximum and agrees with the idea of least informative distribution.

In information theory, Shannon entropy [26] is often used to measure the “amount of information” in a variable.

(9) $$E_{Sh}\;=\;-\sum_{i\;=\;1}^{n}p_{i}.log_{2}\left(p_{i}\right)$$

where *n* is the amount of basic states in a state space and $p_{i}$ is the probability of state *i*. It is clear that the quantity of entropy is always associated with the amount of states in a system. In the framework of DS evidence theory, the uncertain information is represented by both mass functions and the FOD. Deng entropy [9] considers both.

(10) $$E_{Deng}\;=\;-\sum m\left(A\right).log_{2}\frac{m(A)}{\left(2^{\left|A\right|}-1\right)}$$

where |*A*| denotes the cardinality of the focal element *A*. Other works related to entropy under DS framework can be found in the literature [27]. Based on Shannon and Deng entropy, we propose a new belief entropy, which considers *Bel* and *Pl* of mass function, cardinality of focal elements, and number of elements in FOD. The goal of the proposed entropy is to capture the uncertainty of information under DS framework, which are omitted by Shannon and Deng entropy.

(11) $$Proposed\;Entropy,E_{p}\;=\;-\sum \frac{Bel(A)+Pl(A)}{2}.\;log_{2}\left(\frac{Bel(A)+Pl(A)}{2.(2^{\left|A\right|}-1)}.\;\exp^{\left(\frac{|A|-1}{\left|X\right|}\right)}\right)$$

where |*X*| denotes the cardinality of *X*, which represents the number of element in FOD. The exponential factor $\exp^{\left(\frac{|A|-1}{\left|X\right|}\right)}$ in the new belief entropy represents the uncertain information in the number of elements of FOD that has been ignored by Deng entropy. This probability interval considers the lower and upper bounds of evidence that are Bel and Pl, respectively. The new belief entropy which considers Deng entropy and the interval probability can better measure the uncertainty of BPA.

### Properties of Proposed Entropy Function

**Property** **1.** *Mathematically, the value range of the new belief entropy is (0,+∞). According to DS evidence theory, a focal element A consists of at least one element and the limit of its element number is the scale of FOD. FOD consists of at least one element, and there is no maximum limit; thus, the ranges of |A| and |X| are the same, denoted as [1,+∞). The range of a mass function m(A) is (0,1]. Depending on the value of |A|, the believe (Bel) and plausibility (Pl) ranges could be between (0,+∞]. In the proposed entropy equation, where |A|∈[1,+∞) and |X|∈[1,+∞), Bel(A)∈(0,+∞] and Pl(A)∈(0,+∞]. Thus, the range of the proposed entropy can be denoted (0,+∞).*

**Property** **2.** *New belief entropy can degenerate to the Shannon entropy when the mass function is Bayesian. If the mass function m(A) is Bayesian, then BPA is assigned only on single element subset and |A| = 1. In this case, the new belief entropy can degenerate to the following equation, which is exactly equal to Shannon entropy:*

$$=\;-\sum \frac{m_{i}+m_{i}}{2}.log_{2}\left(\left(\frac{m_{i}+m_{i}}{2.(2^{1}-1)}\right).\exp^{\left(1-1)/|X\right|}\right)\;=-\sum m\left(A\right).log_{2}\left(m\left(A\right)\right)$$

**Property** **3.** *Non-negativity. We know that $0<(Bel\left(m_{i}\right)+Pl\left(m_{i}\right))/2<1$. As a result, entropy (m)>0. Only if m(A)=1 and only if A is Bayesian, then Entropy (m)=0. Thus, new entropy satisfies the non-negativity property.*

**Property** **4.** *Consistency with DS theory framework. The new entropy is consistent with the DS theory framework. Thus, it satisfies the consistency with DS theory framework properties.*

**Property** **5.** *Probability consistency. If m is Bayesian, then m(A)=Bel(A)=Pl(A) for all A∈X. Thus, new entropy satisfies the probability consistency property.*

The following example shows the properties of proposed entropy and how it is better at capturing uncertainties compared to Shannon and Deng entropy.

**Example** **5.** *Given a frame of discernment Θ={a,b,c} for a mass function m(a)=m(b)=m(c)=1/3.*

$E_{Sh}\;=\;-\left(\frac{1}{3}log_{2}\frac{1}{3}+\frac{1}{3}log_{2}\frac{1}{3}+\frac{1}{3}log_{2}\frac{1}{3}\right)\;=\;1.585$

$E_{Deng}\;=\;(\frac{1}{3}log_{2}\frac{1/3}{2^{1}-1}+\frac{1}{3}log_{2}\frac{1/3}{2^{1}-1}+\frac{1}{3}log_{2}\frac{1/3}{2^{1}-1}\;=\;1.585$

$E_{P}\;=\;-(\frac{\left(1/3+1/3\right)}{2}log_{2}\frac{\left(1/3+1/3\right)}{2.(2^{1}-1)}.\exp^{\left(\frac{1-1}{3}\right)}\;+\;\frac{\left(1/3+1/3\right)}{2}log_{2}\frac{\left(1/3+1/3\right)}{2.(2^{1}-1)}.\exp^{\left(\frac{1-1}{3}\right)}\;+\;$

$\frac{\left(1/3+1/3\right)}{2}log_{2}\frac{\left(1/3+1/3\right)}{2.(2^{1}-1)}.\exp^{\left(\frac{1-1}{3}\right)})\;=\;1.585$

This showed that the result of the proposed entropy is identical to Shannon entropy and Deng entropy when the belief is only assigned on single elements (or Bayesian).

**Example** **6.** *Given a frame of discernment Θ={a,b,c}, mass function m(a)=m(b)=m(c)=m(a,b)=m(a,c)=m(b,c)=m(a,b,c)=1/7. Bel and Pl values can be calculated for all the BPA, which is shown in Table 2.*

Shannon and Deng entropy is calculated to compare with the values from proposed entropy.

$$\begin{array}{c} E_{Sh}\;=\;-\left(\frac{1}{7}log_{2}\frac{1}{7}+\frac{1}{7}log_{2}\frac{1}{7}+\frac{1}{7}log_{2}\frac{1}{7}+\frac{1}{7}log_{2}\frac{1}{7}+\frac{1}{7}log_{2}\frac{1}{7}+\frac{1}{7}log_{2}\frac{1}{7}+\frac{1}{7}log_{2}\frac{1}{7}\right)\;=\;2.8074 \end{array}$$

$$\begin{array}{c} E_{Deng}\;=\;-\left(\frac{1}{7}log_{2}\frac{1}{7}+\frac{1}{7}log_{2}\frac{1}{7}+\frac{1}{7}log_{2}\frac{1}{7}+\frac{1}{7}log_{2}\frac{1}{3*7}+\frac{1}{7}log_{2}\frac{1}{3*7}+\frac{1}{7}log_{2}\frac{1}{3*7}+\frac{1}{7}log_{2}\frac{1}{7*7}\right)\;=\;3.887 \end{array}$$

$$\begin{array}{c} E_{P}\;=\;-(\frac{5}{2*7}log_{2}\frac{5}{2*7}+\frac{5}{2*7}log_{2}\frac{5}{2*7}+\frac{5}{2*7}log_{2}\frac{5}{2*7}+\frac{9}{2*7}log_{2}\left(\frac{9}{2*3*7}.\exp^{\left(1/3\right)}\right)+\frac{9}{2*7}log_{2}\left(\frac{9}{2*3*7}.\exp^{\left(1/3\right)}\right)+\\ \frac{9}{2*7}log_{2}\left(\frac{9}{2*3*7}.\exp^{\left(1/3\right)}\right)+log_{2}\left(\frac{1}{7}.\exp^{\left(2/3\right)}\right))\;=\;6.79 \end{array}$$

Shannon entropy only considers mass function value and has the lowest entropy. Deng entropy considers both mass function value and cardinality on focal elements. It calculates higher entropy than Shannon. Proposed entropy considers mass function value (central value of probability interval), cardinality of both focal elements, and FOD. It results in the highest entropy value compared to Shannon and Deng. If a FOD consists of 7 elements compared to say 3 elements, intuitively it can be said that the 7-element FOD should have higher entropy because it is less compact. Also, because the proposed entropy considers central value of probability interval $\frac{\left(Bel+Pl\right)}{2}$, it is capturing more uncertainty compared to only mass function. As a result, the proposed entropy function abides by the DS framework and is superior in capturing uncertainty compared to Shannon and Deng entropy.

## 6. Proposed Steps to Eliminate Paradoxes

With increasing use of sensors application in real-time decision making, we need an algorithm which can fuse sensor outputs both in the space domain and the time domain. The goal of the proposed method is to eliminate the paradoxes of the original DS combination rule and work as a decision-level sensor fusion algorithm in both the space and time domains. We are adopting “revision of original evidence before combination” because we do not want to lose the associative and commutative properties of the original DS rule. The proposed method is a distance-based method. It calculates the relative distances between the sensor evidences (classification output). Then, based on average distance, it classifies which sensor output is credible and which sensor output is incredible. Then, it penalizes the incredible sensor output using the novel entropy function so that the incredible sensor has less effect on the fused output. It also rewards the credible sensor input so that the credible sensor carries more weight towards the fused output. At the end, modified evidence is fused using the original DS sensor fusion equation. The following example is used to showcase the steps and to compare the final fused results with works from open literature.

**Example** **7.** *In a multisensor-based target recognition system, assume there are three types of targets to be recognized: {A,B,C}. Suppose there are five sensors. They could be any type of sensors. After data acquisition at a specific moment by five sensors, data are processed and classification IDs are generated. Generated IDs from five sensors are listed as BPAs:*

*Sensor 1: $m_{1}:m_{1}\left(A\right)\;=\;0.41,m_{1}\left(B\right)\;=\;0.29,m_{1}\left(C\right)\;=\;0.30$*

*Sensor 2: $m_{2}:m_{2}\left(A\right)\;=\;0.00,m_{2}\left(B\right)\;=\;0.90,m_{2}\left(C\right)\;=\;0.10$*

*Sensor 3: $m_{3}:m_{3}\left(A\right)\;=\;0.58,m_{3}\left(B\right)\;=\;0.07,m_{3}\left(A,C\right)\;=\;0.35$*

*Sensor 4: $m_{4}:m_{4}\left(A\right)\;=\;0.55,m_{4}\left(B\right)\;=\;0.10,m_{4}\left(A,C\right)\;=\;0.35$*

*Sensor 5: $m_{5}:m_{5}\left(A\right)\;=\;0.60,m_{5}\left(B\right)\;=\;0.10,m_{5}\left(A,C\right)\;=\;0.30$*

This is a classic example of the “one ballot veto” paradox. Bel and Pl values can be calculated for all the BPA, which is shown in Table 3.

**Step 1**: Build a multi-sensor information matrix. Assume, for a multi-sensor system, there are N evidences (sensors) in the frame $\Theta \;=\;\{H_{1},H_{2},\ldots ..,H_{M}\}$ (objects to be detected).
  (12) $$\left(\begin{array}{cccc} m_{1}\left(H_{1}\right) & m_{1}\left(H_{2}\right) & \cdots & m_{1}\left(H_{M}\right)\\ m_{2}\left(H_{1}\right) & m_{2}\left(H_{2}\right) & \cdots & m_{2}\left(H_{M}\right)\\ \vdots & \vdots & \ddots & \vdots \\ m_{N}\left(H_{1}\right) & m_{N}\left(H_{2}\right) & \cdots & m_{N}\left(H_{M}\right) \end{array}\right)=\left(\begin{array}{cccc} 0.41 & 0.29 & 0.3 & 0\\ 0 & 0.9 & 0.1 & 0\\ 0.58 & 0.07 & 0 & 0.35\\ 0.55 & 0.1 & 0 & 0.35\\ 0.6 & 0.1 & 0 & 0.3 \end{array}\right)$$
**Step 2**: Measure the relative distance between evidences. Several distance function can be used to measure the relative distance. They all have their own advantages and disadvantages regarding runtime and accuracy. We have used Jousselme’s distance [28] function. Jousselme’s distance function uses cardinality in measuring distance which is an important metric when multiple elements are present in one BPA under DS framework. The effect of different distance functions (Euclidean, Jousselme, Minkowsky, Manhttan, Jffreys, and Camberra distance function) on simulation time and information fusion can be found in the literature [29]. Assuming that there are two mass functions indicated by $m_{i}$ and $m_{j}$ on the discriminant frame Θ, the Jousselme distance between $m_{i}$ and $m_{j}$ is defined as follows:
  (13) $$DM\left(m_{i},m_{j}\right)\;=\;\sqrt{\frac{1}{2}.\left(m_{i}-m_{j}\right).D.{\left(m_{i}-m_{j}\right)}^{T}}$$
  where $D\;=\;\frac{\left|A\cap B\right|}{\left|A\cup B\right|}$ and |.| represents cardinality.
**Step 3**: Calculate sum of evidence distance for each sensor.
  (14) $$d_{i}\;=\;\sum_{j=1\;\&\;j\neq i}^{N}DM\left(m_{i},m_{j}\right)\;=\;\left(\begin{array}{c} 1.5449\\ 2.9284\\ 1.2311\\ 1.1761\\ 1.1944 \end{array}\right)$$
**Step 4**: Calculate global average of evidence distance.
  (15) $$\bar{d}\;=\;\frac{\sum_{i=1}^{N}d_{i}}{N}\;=\;1.615$$
**Step 5**: Calculate belief entropy for each sensor by using Equation (11), and normalize.

It is interesting to note that, although $m_{3},m_{4},$ and $m_{5}$ have zero *m(C)* values, it has nonzero *Pl* values. As a result, it will consider nonzero *Pl* values of *m(C)* when calculating entropy. $E_{P}\left(m_{1}\right)=1.5664,\;E_{P}\left(m_{2}\right)=0.469,\;E_{P}\left(m_{3}\right)=1.3861,\;E_{P}\left(m_{4}\right)=1.513,\;E_{P}\left(m_{5}\right)=1.483$. Normalize the entropy:

(16) $$\bar{E_{P}\left(m_{i}\right)}\;=\;\frac{E_{p}\left(m_{i}\right)}{\sum E_{p}\left(m_{i}\right)}$$

**Step 6**: The evidence set is divided into two parts: the credible evidence and the incredible evidence. From Equations (14) and (15):
  (17) $$\begin{array}{c} If\;d_{i}\leq \bar{d},m_{i}\;is\;credible\;evidence\\ If\;d_{i}>\bar{d},m_{i}\;is\;incredible\;evidence \end{array}$$

The intuition is that, if an evidence has higher distance than average distance (which is calculated using all the evidences), then probably that evidence is faulty and should be penalized (incredible evidence). If an evidence distance is lower than average, then that evidence is in harmony with other evidence and should be rewarded (credible evidence). Lower entropy means lower uncertainty, and that evidence should be rewarded more for credible evidence. The opposite is true for incredible evidence. Therefore, we needed a function which has large slope as it goes near to zero. Natural log function fits the bill. As a result, the following reward and penalty function is proposed:

(18) $$\begin{array}{c} For\;credible\;evidence,\;Reward\;function\;=-ln(\bar{E_{P}\left(m\right)})\\ For\;incredible\;evidence,\;Penalty\;function\;=-ln(1-\bar{E_{P}\left(m\right)}) \end{array}$$

Using Equations (16)–(18), calculate reward and penalty value for each evidence. $Reward_{1}\;=\;1.4103,\;Penalty_{2}=0.0759,\;Reward_{3}=1.5326,\;Reward_{4}=1.445,\;Reward_{5}=1.4647$.

Normalize reward and penalty values to get evidence weights. $w_{1}=0.2379,\;w_{2}=0.0128,\;w_{3}=0.2585,\;w_{4}=0.2437,\;w_{5}=0.2471$. Obviously, we can observe that there is a high conflict between the evidence $m_{2}$ and other evidences. Therefore, $m_{2}$ is defined as an incredible evidence and has very low weight. Other evidences are supported by each other, so their weights are higher than $m_{2}$.

**Step 7**: Modify the original evidences.
  (19) $$m\left(A\right)\;=\;\sum_{i=1}^{N}m_{i}\left(A\right).w_{i}$$

The resulting modified evidences are *m(A)* = 0.5298, *m(B)* = 0.1477, *m(C)* = 0.0726 and *m(A,C)* = 0.2499.

**Step 8**: Combine modified evidence for (n−1) times (for this example, 4 times) with DS combination rule by using Equations (5) and (6). How to apply the fusion rule is important. For this example, if evidences $m_{1}$ and $m_{2}$ are fused with modified evidence, then $m_{12}\left(A\right)=0.8125,\;m_{12}\left(B\right)=0.0325,\;m_{12}\left(C\right)=0.062\;and\;\;m_{12}\left(A,C\right)=0.093$. Now, to get $m_{123}$, if $m_{12}$ values are fused with $m_{12}$ values using Equations (5) and (6), that would be wrong. To get $m_{123}$, $m_{12}$ values should be fused with the original modified evidence from step 7. It is also evident that, for single elements, if that element has higher value after step 7, it will have highest value after fusing (n−1) times. The higher the value after step 7, the higher the value after fusion.

Table 4 compares the results of the proposed algorithm with other combination methods from open literature for Example 7.

**Figure 2:**
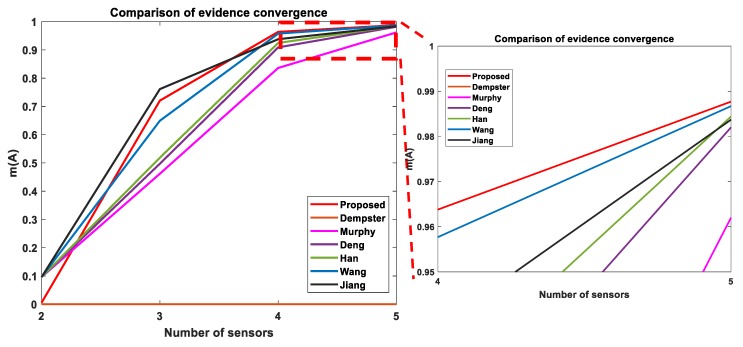
Comparison of convergence of evidence m(A) for Example 7.

As seen from Table 4, when evidences are in high conflict, classical Dempster’s combination rule produces counterintuitive results that are not correct. With increases in number of sensors, Murphy’s simple averaging, Deng’s weighted averaging, and Han’s novel weight averaging, Wang’s weighted evidence and Jiang’s uncertainty measure give reasonable results, although their final combination results are slightly inferior to the outcomes of our proposed approach. Wang et al. [32] showed in his paper that the modified evidences before the fusion steps are m(A)=0.5048,m(B)=0.184,m(C)=0.068, and m(AC)=0.243. Now, the modified evidence for *m(A)* is lower than our proposed method as stated in step 7. Also as explained in step 8, it is unlikely that, after fusing these evidences (n−1) times (4 for this example) using original DS combination rule, the fused $m_{12345}\left(A\right)$ will be higher than our proposed method. Using the evidences presented in Wang’s work, the recalculated fused evidences are presented in Table 4. The proposed method also has the highest convergence rate (rate of m(A) value goes towards 1) after sensor 3. It is reasonable to say that the proposed method overcomes the paradoxes of classical DS rule and produces competitive fusion results compared to that of combination rule available in open literature. Figure 2 shows how fused evidence of m(A) changes with the addition of new sensors and compares multiple methods from the literature. As m(A) is the correct evidence, how it is changing with the inclusion of new sensor evidence is important for justification of the fused result. The proposed method penalizes m(A) when only two sensors are used. As a result, m(A) starts with lower evidence for the proposed method compared to other methods (number of sensors = 2). However, with the inclusion of correct evidences from sensors 3 and 4, *m(A)* converges towards 1 quickly for the proposed method compared to other methods. As *m(A)* evidence converges towards 1, the convergence rate becomes slow for all the methods. A zoomed-in view shows that the proposed method has higher *m(A)* evidence after fusing 5 sensor evidences compared to other methods from the literature.

It can be seen from Example 7 that this method is applicable for any multi-sensor system because fusion occurs after classification ID is created from sensor output. As an example, let us assume multiple cameras are used for object classification. Camera output (video/image) will go through a classifier (example: neural network) for object ID classification. After classification, the output may have similar syntax to Example 7. Then, the proposed method can be applied to find out which sensor is providing erroneous data and to fuse them accordingly.

## 7. Conclusions

In this paper, an eight-step algorithm under DS framework is introduced as an innovative methodology that can be used to better capture uncertainties related to decision-level multi-sensor fusion. A novel entropy function is proposed based on Shannon entropy which takes into account the central value of probability interval and cardinality of both focal elements and FOD. As a result, it is better at capturing uncertainties under DS framework compared to Shannon and Deng entropy. The proposed algorithm calculates distances between multiple evidences (sensors). Based on evidence distance, it rewards the evidences which agree with one another and penalizes the evidences which disagree. The proposed entropy function is used to calculate the weights of the evidences. Conflicting evidences are modified before using them for spatial domain fusion. Classical DS combination rule is used for decision-level sensor fusion; as a result, associative and commutative properties are kept. The proposed method is able to suppress the paradoxes of classical DS combination rule. Detailed examples showed that the proposed method produces competitive convergence rate and fusion accuracy in terms of combining the conflicting evidences in the spatial domain compared to other methods available in the literature.

## Figures and Tables

**Table 1:** *Bel* and *Pl* values for Example 1.

	A	B	C	A,B	A,C	B,C	A,B,C
*Bel(.)*	0.48	0.24	0.08	0.72	0.56	0.32	1.0
*Pl(.)*	0.68	0.44	0.28	0.92	0.76	0.52	1.0

**Table 2:** *Bel* and *Pl* values for Example 6.

	a	b	c	a,b	a,c	b,c	a,b,c
Bel(.)	1/7	1/7	1/7	3/7	3/7	3/7	1.0
Pl(.)	4/7	4/7	4/7	6/7	6/7	6/7	1.0

**Table 3:** *Bel* and *Pl* values for Example 7.

	m(A)	m(B)	m(C)	m(A,C)
$m_{1}$	*Bel* = 0.42, *Pl* = 0.41	*Bel* = 0.29, *Pl* = 0.29	*Bel* = 0.3, *Pl* = 0.3	*Bel* = 0, *Pl* = 0
$m_{2}$	*Bel* = 0, *Pl* = 0	*Bel* = 0.9, *Pl* = 0.9	*Bel* = 0.1, *Pl* = 0.1	*Bel* = 0, *Pl* = 0
$m_{3}$	*Bel* = 0.93, *Pl* = 0.93	*Bel* = 0.07, *Pl* = 0.07	*Bel* = 0, *Pl* = 0.35	*Bel* = 0.93, *Pl* = 0.93
$m_{4}$	*Bel* = 0.9, *Pl* = 0.9	*Bel* = 0.1, *Pl* = 0.1	*Bel* = 0, *Pl* = 0.35	*Bel* = 0.9, *Pl* = 0.9
$m_{5}$	*Bel* = 0.9, *Pl* = 0.9	*Bel* = 0.1, *Pl* = 0.1	*Bel* = 0, *Pl* = 0.3	*Bel* = 0.9, *Pl* = 0.9

**Table 4:** Evidence combination results based on different combination methods for Example 7.

Combination Rule	$m_{1},m_{2}$	$m_{1},m_{2},m_{3}$	$m_{1},m_{2},m_{3},m_{4}$	$m_{1},m_{2},m_{3},m_{4},m_{5}$
Dempster [8]	m(A) = 0, m(B) = 0.8969, m(C) = 0.1031	m(A) = 0, m(B) = 0.8969, m(C) = 0.1031	m(A) = 0, m(B) = 0.8969, m(C) = 0.1031	**m(A) = 0**, m(B) = 0.8969, m(C) = 0.1031
Murphy [21]	m(A) = 0.0964, m(B) = 0.8119, m(C) = 0.0917, m(AC) = 0	m(A) = 0.4619, m(B) = 0.4497, m(C) = 0.0794, m(AC) = 0.0090	m(A) = 0.8362, m(B) = 0.1147, m(C) = 0.0410, m(AC) = 0.0081	**m(A) = 0.9620**, m(B) = 0.0210, m(C) = 0.0138, m(AC) = 0.0032
Deng [30]	m(A) = 0.0964, m(B) = 0.8119, m(C) = 0.0917, m(AC) = 0	m(A) = 0.4974, m(B) = 0.4054, m(C) = 0.0888, m(AC) = 0.0084	m(A) = 0.9089, m(B) = 0.0444, m(C) = 0.0379, m(AC) = 0.0089	**m(A) = 0.9820**, m(B) = 0.0039, m(C) = 0.0107, m(AC) = 0.0034
Han [31]	m(A) = 0.0964, m(B) = 0.8119, m(C) = 0.0917, m(AC) = 0	m(A) = 0.5188, m(B) = 0.3802, m(C) = 0.0926, m(AC) = 0.0084	m(A) = 0.9246, m(B) = 0.0300, m(C) = 0.0362, m(AC) = 0.0092	**m(A) = 0.9844**, m(B) = 0.0023, m(C) = 0.0099, m(AC) = 0.0034
Wang [32] recalculated	m(A) = 0.0964, m(B) = 0.8119, m(C) = 0.0917, m(AC) = 0	m(A) = 0.6495, m(B) = 0.2367, m(C) = 0.1065, m(AC) = 0.0079	m(A) = 0.9577, m(B) = 0.0129, m(C) = 0.0200, m(AC) = 0.0094	**m(A) = 0.9867**, m(B) = 0.0008, m(C) = 0.0087, m(AC) = 0.0035
Jiang [20]	m(A) = 0.0964, m(B) = 0.8119, m(C) = 0.0917, m(AC) = 0	m(A) = 0.7614, m(B) = 0.1295, m(C) = 0.0961, m(AC) = 0.0130	m(A) = 0.9379, m(B) = 0.0173, m(C) = 0.0361, m(AC) = 0.0087	**m(A) = 0.9837**, m(B) = 0.0021, m(C) = 0.0110, m(AC) = 0.0032
Proposed	m(A) = 0.00573, m(B) = 0.96906, m(C) = 0.02522, m(AC) = 0	m(A) = 0.7207, m(B) = 0.1541, m(C) = 0.1178, m(AC) = 0.007	m(A) = 0.9638, m(B) = 0.0019, m(C) = 0.0224, m(AC) = 0.0117	**m(A) = 0.9877**, m(B) = 0.0002, m(C) = 0.0087, m(AC) = 0.0034
